# Involvement of tumor acidification in brain cancer pathophysiology

**DOI:** 10.3389/fphys.2013.00316

**Published:** 2013-11-01

**Authors:** Avinash Honasoge, Harald Sontheimer

**Affiliations:** Department of Neurobiology and Center for Glial Biology in Medicine, University of Alabama at BirminghamBirmingham, AL, USA

**Keywords:** glioma, pH, NHE, brain cancer, solid tumor, acidification

## Abstract

Gliomas, primary brain cancers, are characterized by remarkable invasiveness and fast growth. While they share many qualities with other solid tumors, gliomas have developed special mechanisms to convert the cramped brain space and other limitations afforded by the privileged central nervous system into pathophysiological advantages. In this review we discuss gliomas and other primary brain cancers in the context of acid-base regulation and interstitial acidification; namely, how the altered proton (H^+^) content surrounding these brain tumors influences tumor development in both autocrine and paracrine manners. As proton movement is directly coupled to movement of other ions, pH serves as both a regulator of cell activity as well as an indirect readout of other cellular functions. In the case of brain tumors, these processes result in pathophysiology unique to the central nervous system. We will highlight what is known about pH-sensitive processes in brain tumors in addition to gleaning insight from other solid tumors.

Primary brain tumors stand out amongst solid tumors in both their location and their pathophysiology. The most common and aggressive type of primary brain tumors, glioma, invades brain space while simultaneously destroying surrounding tissue in an attempt to increase brain real estate (Watkins and Sontheimer, 2012). As with other solid tumors, gliomas display enhanced glycolysis and heightened acidification of the tissue interstitium (Vlashi et al., 2011). Unlike other solid tumors, however, gliomas face both brain-specific cellular interactions (Charles et al., 2011) and chemical composition (Irani, 2008). This results in unique pathophysiological consequences. In this review, we will highlight the mechanisms by which brain tumors regulate both their intracellular pH (pH_i_) and also the pH of the surrounding tissue (pH_e_), and how this pH regulation affects tumor pathogenicity.

## pH_i_ regulation

**Figure 1:**
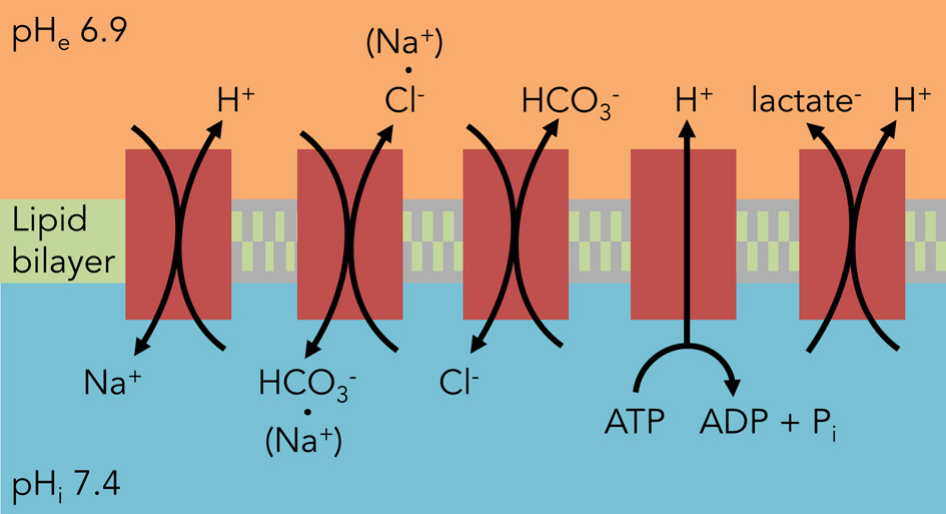
**Major components of pH_i_ regulation in glioma cells.** Unlike astrocytes, glioma cells rely heavily on HCO^−^_3_-independent mechanisms to regulate their pH_i_. The predominant acid extruder is NHE1, a Na^+^/H^+^ exchanger. All of these components except for the H^+^ V-ATPase also contribute to C6 glioma osmotic regulation from the transport of the counter-ion.

Tumor cells constantly struggle to resist the electrochemical gradients of protons, weak acids, and weak bases generally acidifying the cell (Webb et al., 2011; Bevensee and Boron, 2013). Thus a major driving force in understanding tumor acid-base physiology is understanding the transport of protons across the plasma membrane. This transport uses either energy substrates or is coupled to the electrochemically-favorable transport of a second molecule. The following section explains the roles of various H^+^-coupled transporters and exchangers in brain tumor pH_i_ regulation (Figure 1).

Most initial studies on glioma cell pH_i_ regulation used C6 rat glioma cells, which were generated by exposure to *N*, *N′*-nitroso-methylurea and used throughout the 1980s and 1990s as a convenient cell line for studying astrocytic physiology including cytotoxic edema, cerebral ischemia, and volume regulation under osmotic challenge. For a thorough review of the C6 line, please refer to Grobben et al. (2002). These studies often used changes in pH_i_ as a proxy for transport of other ions such as Na^+^, K^+^, and Cl^−^. Only recently did the focus of inquiry shift to human glioma. With this caveat in mind, we will review some older literature that describes pH regulatory systems in C6 followed by newer literature on the biological targets of pH changes in human gliomas.

## Na^+^/H^+^ exchange

Na^+^/H^+^ exchange (NHE) was originally identified in mouse muscle fibers (Aickin and Thomas, 1977) where it was shown to be the major regulator of pH_i_. Owing to a strong inwardly-directed electrochemical gradient for Na^+^, it is ideally suited for proton extrusion, thereby alkalinizing pH_i_ while simultaneously acidifying the interstitium (Figure 1). The first implications of NHE in glioma cells came from studies in C6 rat glioma cells and neuroblastoma x glioma hybrid cells (NG108-15), both used as model systems investigating NHE in adrenergic signaling (Hertel and Staehelin, 1983; Isom et al., 1987a; Nunnari et al., 1987). Later studies involved NHE in a wide variety of signaling pathways (Isom et al., 1987b; Neve et al., 1992). C6 glioma cells were also considered a viable model of glial cells during acidosis and postischemic brain edema, where pH_i_ served as a secondary readout for NHE involvement in osmotic swelling and regulatory volume increase (Jakubovicz et al., 1987; Kempski et al., 1988; Jakubovicz and Klip, 1989; Staub et al., 1994) under mildly acidotic conditions (pH_e_ 6.0–7.0). Later, it was postulated that NHE served to maintain homeostatic pH_i_ at the cost of cell swelling (Kempski et al., 1990; Staub et al., 1990). This exchange was temperature-dependent, with increased activity at higher temperatures (Lui et al., 1995; Mountian et al., 1996). While these studies sought to implicate glial cells in cytotoxic edema, they also hinted at a robust NHE mechanism that would soon be implicated as a hallmark of brain tumors.

The initial reports of NHE in brain tumor cell activity came from C6 glioma spheroids, where H^+^ production under high glucose conditions was diminished by amiloride (Acker et al., 1992). Subsequently, Shrode et al. characterized differences in pH_i_ regulation between C6 glioma cells and astrocytes, with the largest being a lack of Na^+^/HCO^−^_3_ transport in glioma cells (Shrode and Putnam, 1994). McLean et al. were the first to look at various human glioma cell lines and noted a significant elevation in Na^+^/H^+^ exchanger subtype 1 (NHE1) expression, an increase in baseline pH_i_, and an increased reliance on HCO^−^_3_-independent pathways versus primary rat astrocytes (McLean et al., 2000). As the NHE1 blocker amiloride is nonspecific and has off-target effects on glioma cells (Hegde et al., 2004), specific blockade of NHE1 with HOE642 (cariporide) confirmed a tonic activity for the NHE1 exchanger in glioma cell pH_i_ regulation (Glunde et al., 2002). Interestingly, DNA hypermethylation decreases NHE1 expression in oligodendroglioma versus higher-grade gliomas, potentially limiting the growth potential of these lower-grade gliomas (Blough et al., 2012). However, there have not been comprehensive studies of NHE subtypes in gliomas, with one study finding absence of NHE2 and NHE3 expression in C6 glioma cells (Willoughby et al., 2005). More recent studies have hinted at changes in Na^+^/H^+^ exchanger recruitment to the cell surface (Kislin et al., 2009) and spatial organization within the tumor (Grillon et al., 2011).

## Cl^−^/HCO^−^_3_ exchange

C6 glioma cells express both Na^+^-dependent and Na^+^-independent modes of Cl^−^/HCO^−^_3_ exchange (Figure 1). The Na^+^-dependent transport is alkalinizing, while the Na^+^-independent transport is acidifying in response to an intracellular alkalinization (Shrode and Putnam, 1994); these are blocked by the inhibitors 4,4′-diisothiocyano-2,2′-stilbenedisulfonic acid (DIDS) and 4-acetamido-4-isothiocyanatostilbene-2,2-disulfonic acid (SITS) (Kempski et al., 1988; Shrode and Putnam, 1994; Mountian et al., 1996; McLean et al., 2000). Cl^−^/HCO^−^_3_ antiporter activity helps import Cl^−^ ions in tandem with Na^+^ ions from the NHE to serve as osmotic agents for cell swelling in the face of acidosis (Staub et al., 1990; Mountian et al., 1996). The extrusion of HCO^−^_3_ seems to additionally act as a buffer for lactic acid (Staub et al., 1990), a finding discovered while investigating cerebral ischemia but that extrapolates well to the tumor microenvironment. Unlike non-transformed astrocytes, however, it appears glioma cells strongly lean on CO_2_/HCO^−^_3_-independent mechanisms of acid extrusion (McLean et al., 2000).

## H^+^-lactate cotransport

Proton-coupled lactate transporters help rid the cell of both acid and lactate loads simultaneously and thus play vital roles in tumor cellular metabolism and osmoregulation (Figure 1). Lactate efflux was first reported in C6 glioma cells to be a pH-dependent phenomenon, with increased efflux at alkaline pH_e_ (Lust et al., 1975). This transport was reversed in an astrocytoma cell line when the extracellular lactate and proton concentrations were increased (Lomneth et al., 1990; Tomsig et al., 1991), and lactate uptake in glioma cells was saturable at lower concentrations of lactate, indicating a carrier-dependent process (Dringen et al., 1995). This transport can be inhibited by the lactate transport inhibitors quercetin and alpha-cyano-4-hydroxycinnamate (CHC), which when used on C6 glioma cells prevented H^+^-lactate efflux and decreased pH_i_ (Volk et al., 1997). More recently, identification of two specific lactate transporters, MCT1 (Froberg et al., 2001; Mac and Nalecz, 2003; Grillon et al., 2011; Miranda-Goncalves et al., 2013) and MCT4 (Grillon et al., 2011; Miranda-Goncalves et al., 2013) in various types of brain tumors has provided molecular targets for disrupting brain tumor metabolism and pH_i_ regulation.

## Vacuolar-type H^+^-ATPase

Despite predominantly functioning as organellar proton pumps, there is evidence that V-ATPases translocate to the plasma membrane and regulate pH_i_ in brain tumors (Figure 1). V-ATPase inhibitors such as bafilomycin A1 depolarized the membranes of NG108-15 neuroblastoma × glioma hybrid cells (Gerard et al., 1994, 1998) and C6 glioma cells (Philippe et al., 2002). Additionally, this V-ATPase was tonically active and alkalinized C6 glioma cells at physiological pH_i_ (Volk et al., 1998). It should be noted, however, that plasma membrane expression of this proton pump is not limited to gliomas but also occurs in non-transformed astrocytes (Philippe et al., 2002). A more recent study has isolated the a4 isoform of the V0 subunit in human glioma samples, a subunit usually absent in normal human brain that is expressed in the kidney and epididymis (Gleize et al., 2012).

## Aquaporins and carbonic anhydrases

Both of these protein types serve to facilitate a more rapid regulation of pH_i_. Carbonic anhydrases do so by catalyzing the reversible interconversion of CO_2_ + H_2_O and HCO^−^_3_ + H^+^, while aquaporins may be involved in the direct flux of CO_2_ through the plasma membrane (Endeward et al., 2006; Hub and De Groot, 2006). Gliomas predominantly express carbonic anhydrase 9 (CAIX) and aquaporins 1 and 4 (AQP1 and AQP4). Aquaporins have also been shown to play roles in glioma cell adhesion and maintenance of iso-osmolarity during volume regulation via H_2_O flux (McCoy and Sontheimer, 2007). The direct role of these proteins in brain tumor pathophysiology is outside the scope of this review.

## Consequences of pH_i_ regulation

**Figure 2:**
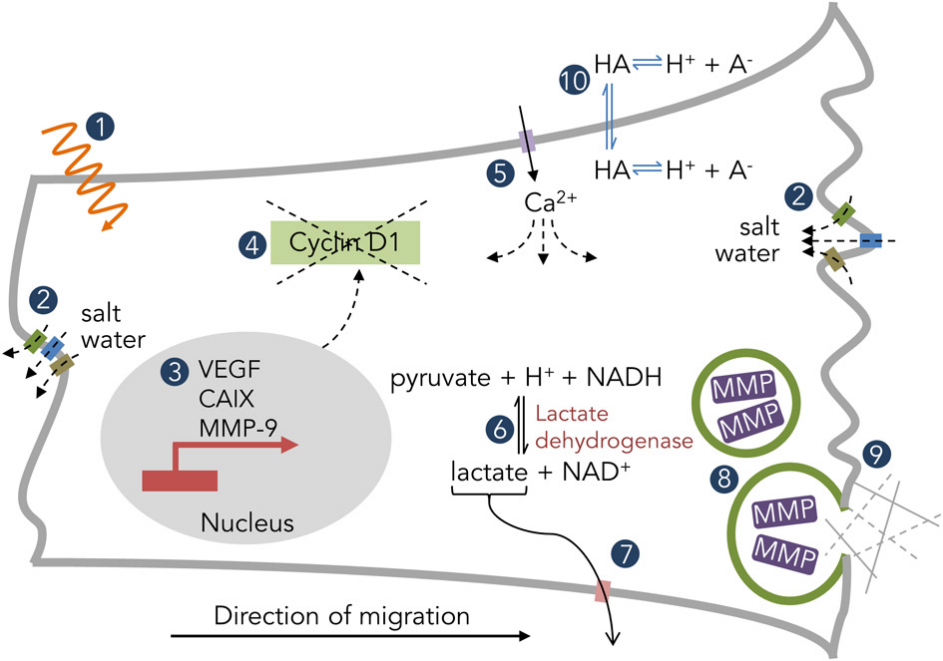
**Examples of pH-dependent physiology in solid tumors.** (1) Radiation efficacy. (2) Salt water flux via K^+^, Cl^−^, and H_2_O channels. (3) Downstream expression patterns of tumorigenic genes. (4) Mislocalization of cyclin D1 and disruption of the cell cycle. (5) Ca^2+^ permeation through ion channels (ASIC, P2X, TRP) and subsequent downstream effects. (6) Metabolic enzyme activity. (7) H^+^-coupled lactate efflux. (8) Vesicular fusion and protease enzymatic activity. (9) Interaction with the extracellular matrix. (10) Distribution of weak acids/bases.

The most direct (and obvious) consequence of pH_i_ regulation is pH_e_ alteration. The aforementioned mechanisms of pH_i_ regulation mostly act as intracellular alkalinizing agents, leading to a large proton efflux into the extracellular space (ECS). The magnitude of pH_i_ and pH_e_ changes depends on buffering capacity, total compartment volume, and molecular diffusivity (Chesler, 2003). These protons do not dissipate readily in the poorly perfused spaces within solid tumors, resulting in pH_e_ heterogeneity and pockets of increased acidity. Therefore, protons may serve as messenger molecules that alter both intratumoral and extratumoral physiology (Figure 2). This section will both review known mechanisms of pH-directed pathophysiology in brain tumors as well as draw lessons from other solid tumor types.

## Ion channel signaling

Brain tumors possess several pH-sensitive ion channels, including acid-sensing ion channels (ASICs), transient receptor potential channels (TRPs), two-pore potassium channels (K2Ps), purinergic receptors (P2XRs), and proton-sensing G-protein coupled receptors (GPCRs). Here we will briefly touch upon channel expression, subtype, and pH-sensitivity as discussed in brain tumor literature. For a broader view of pH-sensitive ion channels and cancer, see Glitsch (2011); for a more in-depth view of ion channels in brain tumors, see Ding et al. (2012).

ASICs are cation-nonspecific (Na^+^, K^+^, and sometimes Ca^2+^) ion channels that are usually opened by low pH_e_ and are transiently active. ASICs 1 and 2 have been shown to be expressed in human glioma cells (Berdiev et al., 2003), with sensitivity to psalmotoxin 1 in addition to amiloride (Bubien et al., 2004). The Na^+^ current derived from glioma ASIC expression contributes to their volume regulation (Ross et al., 2007) and migration (Vila-Carriles et al., 2006; Kapoor et al., 2009). Interestingly, a hybrid of ASIC and epithelial sodium channel (ENaC) subunits creates a basally active conductance (Kapoor et al., 2011) that affects glioma cell migration and cell cycle progression (Rooj et al., 2012). This hybrid channel is recruited to the plasma membrane in the face of acidic pH_e_ as found in the tumor core (Kapoor et al., 2011). Under acidotic conditions, the role of ASIC1a and ASIC2a seems paradoxical: ASIC1a knockdown prevents Ca^2+^-mediated injury (Weng et al., 2007), while ASIC2a knockdown aggravates it (Liu et al., 2011).

TRPs are also cation-nonspecific channels, whose pH-sensitivities play a role in the proton-heavy environments of taste buds, pain receptors, and cancer cells. In brain tumor cells, the expression of TRPC channels has been especially tied to Ca^2+^ influx mediating changes in cell morphology and movement. This includes cytokinesis (Bomben and Sontheimer, 2008, 2010), Ca^2+^ mobilization (Nakao et al., 2008; Chigurupati et al., 2010), and cell migration (Chigurupati et al., 2010; Bomben et al., 2011). Unlike TRPC channels, TRPV channel expression tends to negatively affect glioma cells, leaving them vulnerable to capsaicin-induced apoptosis (Amantini et al., 2007) and chemotherapeutic cytotoxicity (Nabissi et al., 2013), as well as promoting differentiation (Morelli et al., 2012). Interestingly, neural precursor cells (NPCs) release endogenous TRPV agonists that prevent glioma cells from attacking the juvenile brain (Stock et al., 2012), a phenomenon that is lost with a loss of NPCs during aging.

P2XRs are ATP-gated cation channels, while P2YRs are purinergic-coupled GPCRs. Together, they are most extensively studied for their involvement in Ca^2+^ flux in glioma cell signal transduction, tumor progression, and cell death. In general, these receptors are proton-potentiated (Glitsch, 2011). For a comprehensive review of purinergic signaling in glioma cells, refer to Barañska (2013); for the pH-sensitivities of these channels, refer to Stoop et al. (1997), Gerevich et al. (2007).

K2P channels and pH-sensitive GPCRs have not been studied extensively in brain tumor tissue, though their roles in other cancers have been elucidated (Sin et al., 2004; Innamaa et al., 2013). The K2P members TASK-1 and TASK-3, pH-sensitive background K^+^ channels, have functional expression in human medulloblastoma cells (Ernest et al., 2010) and have been functionally implicated in glioma cell survival (Meuth et al., 2008). The pH-sensitive GPCRs OGR1 and G2A are also expressed in human medulloblastoma cells and regulate intracellular Ca^2+^ signaling in response to extracellular pH (Huang et al., 2008).

## Volume regulation and cell movement

Gliomas are highly invasive, quickly seeding the brain with satellite tumors. This is especially impressive in the crowded brain space and requires a coordinated effort of cell shrinkage, process extension, and path-clearing. Volume regulation is a vital component of the first two functions and requires salt water flux (cation + anion + H_2_O; Figure 2) (Sontheimer, 2008; Watkins and Sontheimer, 2011). Two of the most well-studied ion channels in glioma cell migration, BK for K^+^ ions and ClC-3 for Cl^−^ ions, are pH-sensitive. More specifically, low pH_e_ blocks both channels, while low pH_i_ stimulates BK channels, all within a physiological range of pH 6–8 (Avdonin et al., 2003; Brelidze and Magleby, 2004; Matsuda et al., 2008, 2010). Additionally, as described beforehand, proton flux through glioma cells is directly tied to osmotically-active Na^+^ and lactate, which both then contribute to volume regulation (Jakubovicz and Klip, 1989; Staub et al., 1990). Thus, protons can both directly and indirectly contribute to glioma cell volume regulation, which then affects cell movement.

Proton concentrations also both alter shape and orient tumor cells. This has been most thoroughly studied regarding NHE in melanoma cells, though gliomas similarly possess increased NHE1 activity versus their non-transformed counterparts (McLean et al., 2000) with specific microdomain localization (Willoughby et al., 2005). In melanoma cells, NHE1 creates a local pH gradient that dominates the bulk solution pH and orients the cells via pH-dependence of integrin α2β 1 stickiness (Stock et al., 2005; Stuwe et al., 2007; Martin et al., 2011). Intracellularly, NHE1 also organizes the cytoskeleton of cells. For instance, the Rho GTPase Cdc42 recruits NHE1 to the leading edge of the cell, increasing leading edge pH_i_ and activating Cdc42 via a guanine nucleotide exchange factor, thus, maintaining polarized cytoskeletal growth (Frantz et al., 2007). Similarly, cortactin phosphorylation recruits NHE1 to the invadopodium, where it alkalinizes pH_i_ and induces actin polymerization via cortactin release of cofilin (Magalhaes et al., 2011), thus playing an integral role in invadopodium protrusion/retraction cycling. NHE1 is further activated at the invadopodium by p90 ribosomal S6 kinase under hypoxic conditions (Lucien et al., 2011).

## Tissue destruction

Brain tumors also cause direct destruction of surrounding tissue, including both neuronal death via glutamate excitotoxicity (Ye and Sontheimer, 1999) and degradation of the extracellular matrix via metalloproteinases (MMPs) and other proteases (Nakada et al., 2003). It is well-established that protease activity is pH-dependent (Fasciglione et al., 2000; Gioia et al., 2010). Additionally, however, acidic pH_e_ both induces MMP-9 expression (Kato et al., 2005) and enhances the rupture of protease-containing vesicles (Taraboletti et al., 2006), hinting at acidosis driving tumor invasion (Figure 2). RNAi inhibition of MMP-9 and the protease cathepsin B dramatically reduced tumor pathogenicity of gliomas both *in vitro* and *in vivo* (Lakka et al., 2004).

Here again NHE1 plays a role. While in the intracellular compartment NHE1-dependent alkalinization coordinates tumor cell invasion, in the extracellular compartment the consequent acidification is essential for proteolysis of the extracellular matrix (Busco et al., 2010). Interestingly, preventing ion translocation through NHE1 alone was sufficient to alter the gene profile of mammalian fibroblasts, including a decrease in MMP-9 expression (Putney and Barber, 2004).

Finally, the excitotoxic process is itself pH-dependent. Excitatory amino acid transporter 2 (EAAT-2), expressed in low-grade brain tumors (De Groot et al., 2005), cotransports protons along with glutamate and thus is pH-dependent (Vandenberg et al., 1998). The alanine-cysteine-serine transporter 2 (ASCT2) also transports glutamate, and it has shown pH-dependence in C6 glioma cells (Doliñska et al., 2003). Lastly, the NMDA glutamate receptors in part responsible for neuronal excitotoxicity are inhibited by protons (Traynelis and Cull-Candy, 1990).

## Metabolic activity

Gliomas, like most other cancers, demonstrate the Warburg effect—a preference for glycolysis over oxidative phosphorylation even in the presence of ample oxygen. This leads to increased intracellular lactate buildup, which is cleared via the cotransport of lactate and protons via MCTs (Figure 2). Thus, inhibition of these cotransporters via drug or decreased pH_e_ both decreases pH_i_ (Volk et al., 1997) and increases intracellular lactate levels (Lomneth et al., 1990). As many glycolytic enzymes prefer the slightly alkaline pH_i_ of glioma cells—lactate dehydrogenase displays maximal activity at pH_i_ 7.5 while phosphofructokinase 1 works best between pH_i_ 7.0 and 7.5 (Webb et al., 2011)—there is an intimate coupling of glioma pH regulation and cell metabolism. This connection likely governs the expression patterns of pH-associated proteins across the glioma mass (Grillon et al., 2011).

## Cell signaling

It is often difficult to separate the consequences of the various conditions found within a tumor; levels of CO_2_, O_2_, lactate, waste products, and pH distribute through the tumor heterogeneously, and all can influence the cellular phenotype. A few studies have specifically implicated acidosis in an alteration of glioma cell state. For instance, a pH_e_ of 6.6 upregulated VEGF mRNA and protein expression in human GBM cells via the ERK1/2 MAPK signaling cascade (Xu et al., 2002). Acidosis (pH_e_ 6.5) also maintained the stemness of glioma cells as determined both by stem cell markers and cellular phenotype via hypoxia inducible factor 2α (HIF2α) signaling (Hjelmeland et al., 2011). Acyl-CoA synthetase 5 (ACSL5) promotes glioma cell survival under low pH_e_ conditions through midkine (MDK) signaling (Mashima et al., 2009). CA IX, known to be upregulated during times of hypoxia via the HIF1α pathway (Wykoff et al., 2000), is also upregulated by low pH_e_ independent of hypoxia in GBM cells via the same pathway (Ihnatko et al., 2006). Finally, very low pH_e_ (6.0) arrested glioma cells in the G_1_ phase of the cell cycle as a downstream result of cyclin D_1_ mislocalization (Figure 2) and degradation in T98G human glioma cells (Schnier et al., 2008).

## Therapy sensitivity

A heterogeneous pH environment creates a moving target for both radiation and chemotherapeutics. Weak base and weak acid drugs find themselves confined to either the intracellular or extracellular spaces (Figure 2), depending on pH_e_ and pH_i_, in a phenomenon known as “ion trapping” (Raghunand and Gillies, 2000). This can result in heterogeneous drug potency across the tumor mass, and has led to efforts to either acidify or alkalinize the tumor in an attempt to localize chemotherapeutics to either the intra- or extracellular compartment. In gliomas, mild acidosis inhibits cell growth while protecting cells from chemotherapeutic cytotoxicity (Reichert et al., 2002). Attempts have been made to artificially alkalinize solid tumors with NaHCO_3_-induced metabolic alkalosis to enhance weak base uptake (Raghunand et al., 2001), though none of these studies have yet been performed in brain tumors. pH also can affect the radiosensitivity of cells (Bosi et al., 1991), though its effect on glioma cells appears inconsistent (Reichert et al., 2002).

With highly buffered ions such as Ca^2+^ and protons, nanomolar changes in the free ion concentration equate to severalfold shifts and drastic changes in central nervous system (CNS) signaling. It is this context that separates brain tumors from other solid tumors—the pathophysiological implications of large pH heterogeneity in a susceptible environment are greater than in many other, more robust organs. This also leads to great opportunity—brain tumors lean heavily on pH regulation to continue their growth and invasion, and thus disruption of proton transport could devastate tumor function while leaving normal tissue relatively unharmed.

### Conflict of interest statement

The authors declare that the research was conducted in the absence of any commercial or financial relationships that could be construed as a potential conflict of interest.
